# Freshwater fish diversity in the western Amazon basin shaped by Andean uplift since the Late Cretaceous

**DOI:** 10.1038/s41559-023-02220-8

**Published:** 2023-10-19

**Authors:** Lydian M. Boschman, Luca Carraro, Fernanda A. S. Cassemiro, Jorad de Vries, Florian Altermatt, Oskar Hagen, Carina Hoorn, Loïc Pellissier

**Affiliations:** 1 Department of Environmental System Science, ETH Zürich, Zürich, Switzerland; 2 Swiss Federal Research Institute WSL, Birmensdorf, Switzerland; 3 Department of Evolutionary Biology and Environmental Studies, University of Zürich, Zürich, Switzerland; 4 Department of Aquatic Ecology, Eawag, Swiss Federal Institute of Aquatic Science and Technology, Dübendorf, Switzerland; 5 Department of Ecology, Universidade Federal de Goiás, Goiânia, Goiás, Brazil; 6 Forest Ecology and Forest Management Group, Wageningen University, Wageningen, the Netherlands; 7 German Centre for Integrative Biodiversity Research (iDiv) Halle–Jena–Leipzig, Leipzig, Germany; 8 Institute for Biodiversity and Ecosystem Dynamics, University of Amsterdam, Amsterdam, the Netherlands; 9 Present Address: Department of Earth Sciences, Utrecht University, Utrecht, the Netherlands

**Keywords:** Biodiversity, Solid Earth sciences, Biodiversity

## Abstract

South America is home to the highest freshwater fish biodiversity on Earth, and the hotspot of species richness is located in the western Amazon basin. The location of this hotspot is enigmatic, as it is inconsistent with the pattern observed in river systems across the world of increasing species richness towards a river’s mouth. Here we investigate the role of river capture events caused by Andean mountain building and repeated episodes of flooding in western Amazonia in shaping the modern-day richness pattern of freshwater fishes in South America, and in Amazonia in particular. To this end, we combine a reconstruction of river networks since 80 Ma with a mechanistic model simulating dispersal, allopatric speciation and extinction over the dynamic landscape of rivers and lakes. We show that Andean mountain building and consequent numerous small river capture events in western Amazonia caused freshwater habitats to be highly dynamic, leading to high diversification rates and exceptional richness. The history of marine incursions and lakes, including the Miocene Pebas mega-wetland system in western Amazonia, played a secondary role.

## Abstract (web summary)

Reconstructing river networks over the past 80 Ma reveals the role of Andean uplift in creating a dynamic habitat leading to increased fish species diversity in western Amazonia.

## Main

**Fig. 1:**
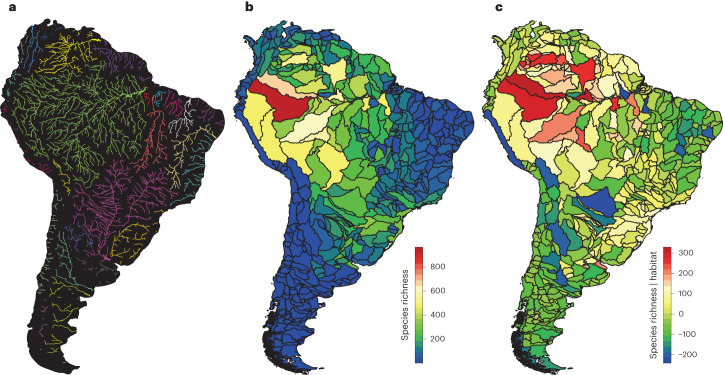
Major river basins and freshwater fish species richness in South America. **a**, Major rivers and drainage basins of South America. HydroRIVERS data are from the HydroSHEDS database^49^; rivers are shown that drain a surface area of >4,000 km^2^. **b**, Freshwater fish species richness per sub-basin (level 5 HydroBASINS^49^), reproduced from data from ref. ^4^. **c**, Residuals of species richness per sub-basin, after correcting for habitat size (that is, water volume; see Methods).

South America is home to the highest freshwater fish biodiversity on Earth^1,2^, and the hotspot of species richness is located in the western Amazon basin (Fig. 1)^3,4^. The location of this hotspot is enigmatic, as it is inconsistent with the pattern observed in river systems across the world of increasing species richness towards a river’s mouth^5,6^. Biodiversity patterns in riverine habitats are thought to be caused by the inherent properties of dendritic river networks, which drive connectivity and shape both biotic and abiotic conditions in riverine landscapes, such as river discharge, habitat complexity or flow variability^7–9^. But biodiversity is built up over millions of years of evolutionary time, during which dendritic connectivity is far from static. Deep-time palaeogeographic, palaeoclimatic and sea level changes can drastically alter the structure and connectivity of river systems through drainage rearrangements, including river capture events (when a stream taps and consequently captures the discharge of a neighbouring stream) or even drainage reversals. These drainage rearrangements change the habitats of the organisms that live in river systems and drive fundamental evolutionary processes. However, net effects of drainage rearrangements on biodiversity are complex: they merge previously isolated habitats and populations, thereby facilitating dispersal, geographical range expansion and gene flow, which increases local diversity initially, but decreases rates of speciation and extinction. At the same time, they separate previously connected habitats, leading to an increase in genetic isolation and an increase in rates of speciation and extinction^10–12^. As a result, palaeogeographic and palaeoclimatic history is expected to have left an imprint on richness patterns observed in river systems today^3^, but assessing this imprint is not straightforward.

In the Amazon River basin, dendritic structure and connectivity have varied through time as a result of the gradual uplift of the Andes since ~80 Ma and the repeated flooding of large areas of western Amazonia. Phylogenetic and palaeontological datasets indicate that the fish fauna of the Amazon region originated during the Late Cretaceous, and provide no evidence for substantial changes in speciation rates through time nor evidence of major extinction events^13–15^. Instead, these datasets show that diversity in Amazonia is the result of a prolonged history of net diversification in which speciation rates exceeded extinction rates^10,13,14,16^. Given the complexity of the effects of drainage rearrangements and the lack of major speciation or extinction events, the diversification history of South American freshwater fish fauna cannot be explained through classic correlative approaches aimed at linking speciation or extinction events to palaeogeographical events. Instead, it requires an approach in which the ambiguous effects of drainage rearrangements can be assessed in a process-based way, and in which palaeogeographic history is not viewed as a series of ‘instantaneous’ (in the context of geological time, within a million years) events, but rather, as continuous and gradual change. Here we investigate the role of Andean mountain building and Amazonian lake system dynamics over the past 80 Ma in shaping the modern-day richness pattern of freshwater fishes in South America. To this end, we reconstruct spatial changes in freshwater habitat connectivity through geological time, and perform modelling experiments focusing on the mechanisms of dispersal, allopatric speciation and extinction on a dynamic landscape of rivers and lakes.

## Palaeogeographic history of South America

Andean mountain building initiated in the Late Cretaceous, at ~100 Ma in Patagonia, ~80 Ma in the Central Andes of Bolivia and Peru, and ~70 Ma in the ranges of Ecuador^17^. During the Late Cretaceous and Palaeocene, the northwestern corner of the South American continent was mostly covered by shallow seas^18^. The bulk of the northern South American rivers flowed towards this northwestern corner, collecting water and sediments from the Brazilian and Guianan shields and the incipient Central Andes before draining into the Caribbean Sea and Pacific Ocean^19^. During the Eocene and Oligocene, uplift migrated to regions farther north^20^, and a continuous continent-scale mountain range was established by the beginning of the Miocene, albeit substantially smaller in width and lower in elevation compared with the modern orogen^21^. The topography in the Northern Andes that was established during the Oligocene–Miocene blocked drainage towards the Pacific and additionally resulted in the formation of a foreland basin^22^. As a result, the Miocene western Amazon basin was characterized by a system of mountain-parallel rivers, lakes and wetlands named the Pebas system, which drained towards the north into the Caribbean Sea^19,23,24^. In eastern Amazonia, the precursor of the modern Amazon River was already present, draining into the Atlantic Ocean^23–25^. Continued uplift in the Northern Andes in the late Miocene and Pliocene produced erosional material forming ‘megafans’ along the eastern slopes of the mountains^26^. This erosion material gradually filled the Miocene foreland basins of western Amazonia, leading to the disappearance of the wetlands, and around ~9 Ma, to the establishment of the transcontinental west-to-east flowing Amazon River^23–25^. For palaeogeographic sketch maps illustrating the evolution of the Andes and Amazon River throughout the Cenozoic, see plates 14–16 in ref. ^19^.

## Results

### Drainage network reconstruction

We generated river networks by combining a river reconstruction algorithm with a recently developed reconstruction of Andean mountain building since 80 Ma (ref. ^21^), consisting of a series of 80 palaeo-digital elevation models (palaeoDEMs, one per million-year time step), at a 0.1° spatial resolution. The river reconstruction algorithm establishes drainage directions for every cell based on the steepest possible descent between neighbouring grid cells, producing a drainage network matching a given topography. Additionally, we incorporated the configurations of the Miocene Pebas wetlands system and other marine incursions and lakes based on other studies^19,27^. We computed drainage networks for three palaeogeographic scenarios designed to test the relative roles of the two main aspects of riverine landscape evolution in Amazonia: (1) mountain building and the consequent drainage rearrangements; and (2) lake system dynamics, which includes the formation and disappearance of lakes (suitable habitat), and marine incursions (non-suitable habitat). Marine incursions modify the location of the shoreline, thereby altering the locations of river outlets. Scenario A includes lake system dynamics but excludes mountain building (that is, topography is identical to modern in all times steps); scenario B includes mountain building but excludes lake system dynamics; and scenario C includes both mountain building and lake system dynamics. Topographic changes east of the Andes are considerably smaller than in the Andes but nonetheless non-zero^28,29^. However, as no quantitative elevation reconstruction is available (data are scarce), we keep the landscape east of the Andes constant. As a result, very little river reorganizations occur in the eastern half of the continent (only those resulting from marine incursions and lake system dynamics), and we therefore refrain from comparing absolute values of habitat change and, in the next step, modelled species richness across the continent. Instead, we focus on the potential mechanisms of species richness development in western Amazonia as a result of palaeogeographic change.

**Fig. 2:**
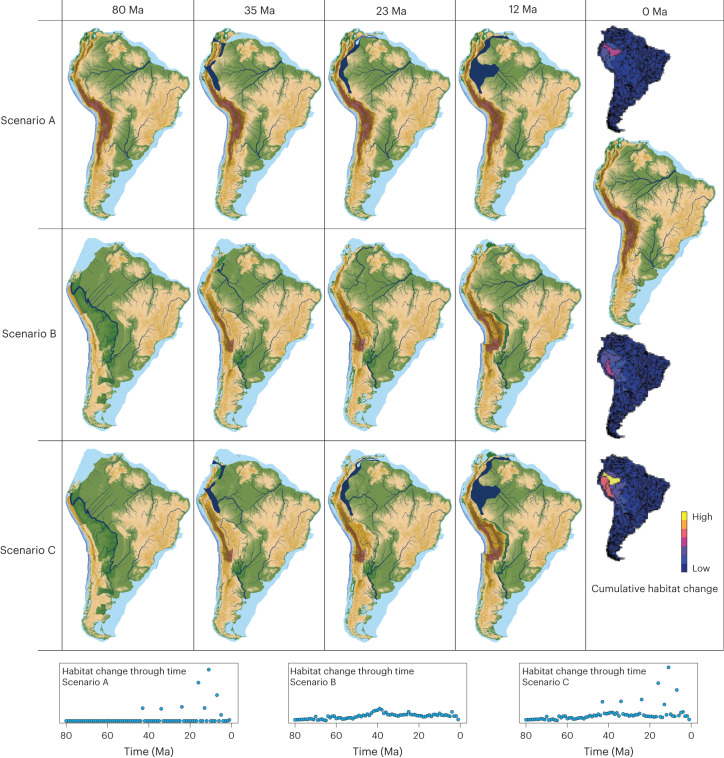
River networks for the three palaeogeographic scenarios. Scenario A: excluding mountain building, including lake system dynamics; scenario B: including mountain building, excluding lake system dynamics; and scenario C: including both mountain building and lake system dynamics. In scenario A, the transcontinental west-to-east flowing Amazon River is present in all time steps, except for during the culmination of the Pebas wetlands system (15–11 Ma), when western Amazonia drained into the Pebas lake, and subsequently, into the Caribbean Sea. Scenarios B and C are identical for early time steps (80–43 Ma), before the presence of marine incursions and lakes, and very similar for 42–24 Ma, in which western Amazonia drained towards the north(west), and eastern Amazonia towards the east. Scenarios B and C start deviating substantially from 23 Ma onwards. In scenario B, the transcontinental west-to-east flowing Amazon River is established at 21 Ma. In scenario C, western Amazonia continues to drain towards the north(west) for 23–11 Ma, and only at 10 Ma, after the disappearance of the Pebas system, is the transcontinental Amazon River established. Cumulative habitat change represents the number of grid cells changing from suitable (river or lake) to unsuitable (land or marine) or vice versa. The lower panels show habitat change plotted through time (change from one time step to the next) and maps in the right panel (0 Ma) show cumulative habitat change over time per sub-basin. Absolute values are meaningless and are omitted.

Results from the river reconstruction algorithm (Fig. 2) indicate that drainage networks changed substantially throughout the past 80 Ma. In all scenarios, these changes occurred primarily in the western Amazon basin, whereas river networks along and in the northernmost and southern parts of the Andes were less dynamic (Fig. 2). This difference somewhat reflects the difference in uplift rate along the Andes (high in the Northern and Central Andes, low in Patagonia), but the absence of high cumulative changes on the flanks of the Central (southern Peru, Bolivia, northern Chile) and northernmost Andes (Colombia, Venezuela) is striking.

Changes in habitat resulting from lake dynamics are larger but less frequent compared with the much more frequent but smaller changes due to mountain building (Fig. 2). Taking into account both surface uplift in the Andes and lakes in the Amazon basin (scenario C), the model produces river morphologies through time that accurately match first-order river reconstructions from sedimentological and palaeontological data, as summarized in the section Palaeogeographic history of South America.

### Biodiversity dynamics

Using the mechanistic biodiversity modelling engine gen3sis (general engine for eco-evolutionary simulations)^30^, we run experiments of biodiversity dynamics over the three palaeogeographic scenarios through the simulation of three mechanisms: dispersal, speciation and extinction. The dispersal rate (distance per time step) determines how far species populations disperse through suitable habitat cells. Speciation occurs through allopatry: when two populations of the same species become disconnected through changes in habitat connectivity, they become two different species. Extinction occurs when all cells in the range of a species change from suitable (river or lake) to unsuitable habitat (land or marine). The simplification of speciation (that is, only through allopatry) is warranted, as assemblages of South American fish species are known to be polyphyletic, meaning that locally coexisting species are rarely each other’s close relatives and geographic ranges of sister species rarely overlap^31–38^. This implies that the origin of these species can primarily be attributed to allopatric speciation, that is, the evolution of a new species as a result of isolation of two populations of a species, and that sympatric speciation, that is, the evolution of a new species from a surviving ancestral species while both inhabit the same geographical range, played a negligible role in the diversification history of South American fishes^31,36,39,40^.

**Fig. 3:**
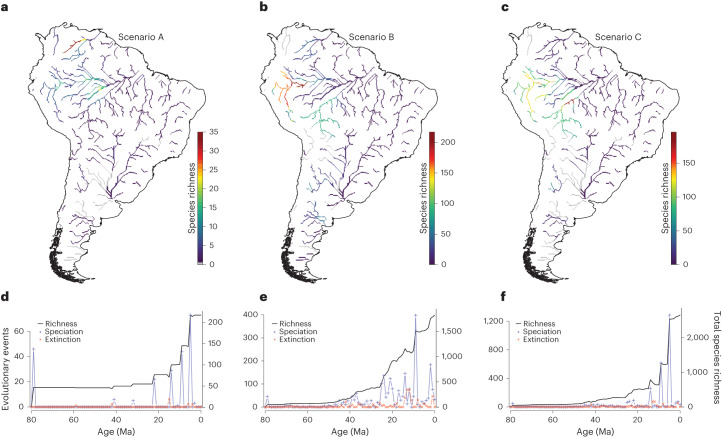
Simulation results. Scenarios as in Fig. 2; *d* = 44.4, div = 2, rs = 200. **a**–**c**, Simulated species richness per grid cell for scenarios A (**a**), B (**b**) and C (**c**). **d**–**f**, Cumulative species richness and speciation and extinction events through model time for scenarios A (**d**), B (**e**) and C (**f**).

**Extended Data Fig. 1:**
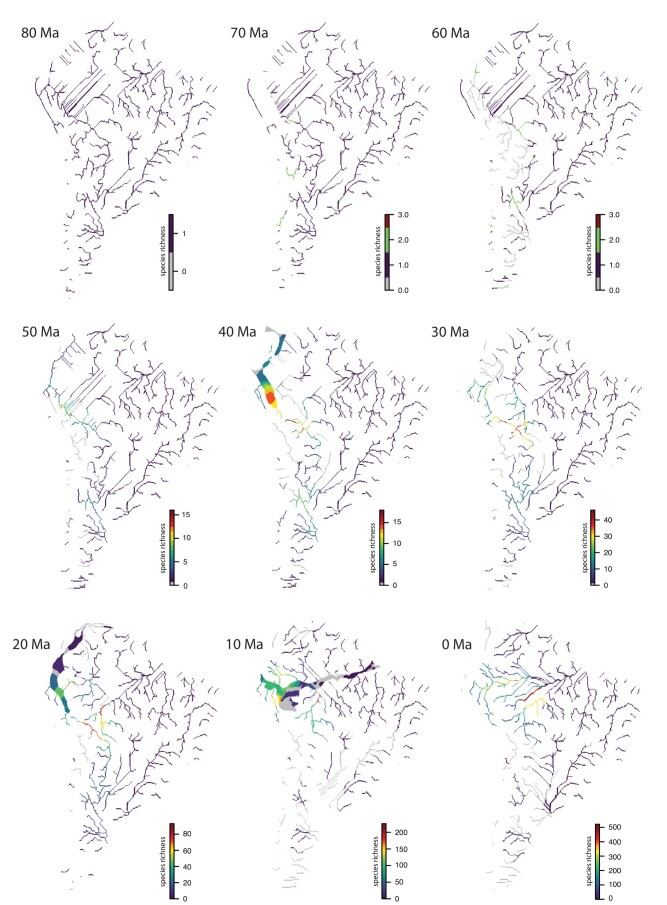
Example of a gen3sis simulation. Snapshots at 80, 70, 60, 50, 40, 30, 20, 10 and 0 Ma; results of richness per sub-basin shown in Fig. 3c.

**Extended Data Fig. 2:**
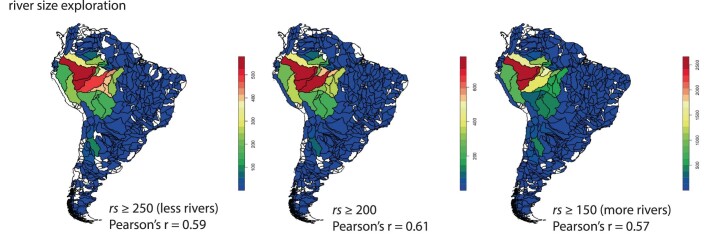
Parameter exploration: river size (*rs)*. *d* = 5, *div* = 2.

**Extended Data Fig. 3:**
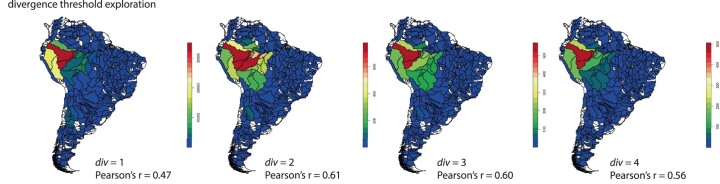
Parameter exploration: divergence threshold (*div)*. *d* = 5, *rs* = 200.

**Extended Data Fig. 4:**
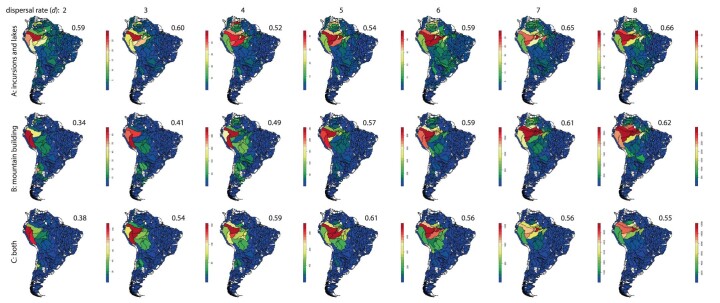
Parameter exploration: dispersal rate (*d)*. *div* = 2, *rs* = 200. Numbers are Pearson’s r.

The biodiversity experiments (Extended Data Fig. 1) consistently produce richness patterns with a hotspot in western Amazonia, irrespective of model parameters (Extended Data Figs. 2–4) and palaeogeographic input scenario (Fig. 3). The experiments indicate that the high species richness in western Amazonia can be attributed to high speciation rates caused by either the gradual uplift of the Central and Northern Andes, or the history of flooding producing wetlands and lakes in western Amazonia, or both. In other words, the species richness hotspot in western Amazonia can be explained by its deep-time palaeogeographic history. Interestingly, the total species richness in reconstruction scenario C is higher than the sum of total species richness in scenarios A and B (Fig. 3d–f), indicating that in the model, interactions between the two components of the palaeogeographic history (mountain building and lake dynamics) intensify the diversification that can be explained by each component individually. However, the much higher number of speciation events resulting from Andean uplift (Fig. 3e) suggests that this has been the primary driver of diversification of freshwater fishes in Amazonia, while the history of flooding played a secondary role.

## Discussion

**Extended Data Fig. 5:**
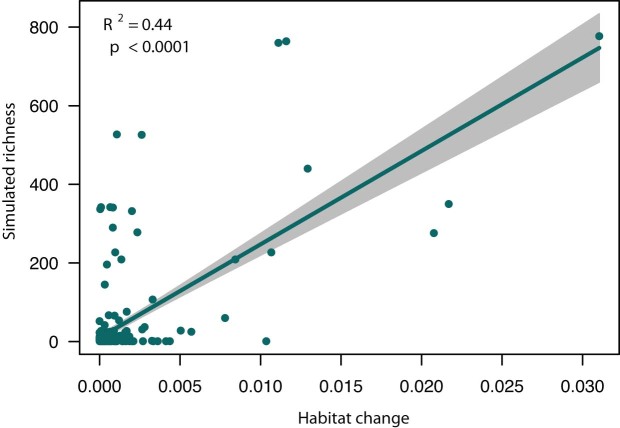
Simulated richness versus cumulative habitat change of sub-basins. Cumulative habitat change per sub-basin (shown in Fig. 2) is a measure of the amount of cells changing from suitable to unsuitable habitat, or vice versa, summed for all 80 time steps. The trendline is obtained using linear regression; the error band is the 95% confidence interval; p < 2.2e-16.

This study highlights the role of drainage rearrangements resulting from topographic change in riverine biodiversity dynamics, and shows that frequently changing river networks promote the accumulation of diversity (Extended Data Fig. 5). Our modelling experiments suggest that the net effect of drainage rearrangements on diversity is positive: genetic drift of isolated populations dominates over gene flow between merged populations, promoting allopatric speciation. We present a method of reconstructing river reorganizations given a quantitative reconstruction of gradual topographic change. This method yields many small but frequent drainage rearrangements in and along the flanks of the Ecuadorian and Peruvian Andes and in western Amazonia. Interestingly, despite uplift rates being similar in the Northern (Colombia, Venezuela) and Central Andes (southern Peru, Bolivia, northern Chile), reconstructed cumulative change in river networks in these regions is substantially smaller (Fig. 2), leading to lower modelled species richness, in line with data. This result reveals the importance of considering small but frequent river reorganizations from gradual topographic change—and thus the method for their reconstruction presented here—which had thus far been neglected as they do not leave a record in the sedimentary archive. This is in contrast to rare, larger river capture events farther downstream^11^ or drainage reversals^25^, which could be reconstructed from sedimentary data.

The river generation algorithm produced a major drainage reversal in the western Amazon basin at around 10 Ma, which coincides well with the age of a large biotic interchange between the western and eastern Amazon basins inferred from phylogenetic data^41^, and the age of transcontinental Amazon River formation inferred from geological data. The latter is based on sedimentological data from exploration wells within the Amazon submarine fan in the Atlantic Ocean, which indicate that sediments of Andean origin reached the Atlantic coast for the first time between 9.4 and 9 Ma (refs. ^23,24,42,43^). Despite establishment of the transcontinental river around 10 Ma, no major changes to the river structure and constant biotic interchange since then, diversity in the Amazon River has not established a ‘normal’ gradient of increasing richness towards the river’s mouth. We suggest that continuous and ongoing landscape change in western Amazonia due to continuous and ongoing uplift in the Andes makes Amazonia the primary area of species origination, resulting in a west-to-east colonization pattern and a persistently undersaturated eastern Amazonia.

Here we have investigated the effects of spatial changes in freshwater habitat connectivity through geological time on biodiversity dynamics. Our findings illustrate the importance of considering deep-time palaeogeographic evolution in studying macroecological patterns. This work builds on previous recognition of the importance of mountain building and landscape dynamics in shaping biodiversity in general^44–46^, and that of river captures on freshwater diversity in particular^47,48^. Previous work on freshwater fish species in South America^3^ had already established the unexpected gradient in species richness along the Amazon River, and these authors attributed this pattern to historical processes. Moreover, another study^4^ recently presented phylogenetic and biogeographic analyses on the history of evolutionary rates and dispersal routes, and these authors again highlighted the importance of palaeogeographic events in shaping current biodiversity patterns. We add to this a mechanistic perspective, which allows assessing the effects of the gradual changes in landscape that affect dispersal, speciation and extinction, and thus diversity, in various ways, thereby going beyond correlative and statistical approaches used in previous work. Using a combination of phylogenetic and mechanistic modelling approaches is the next step in solving biogeographical and macroecological research problems and deciphering the role of palaeogeographic history in shaping biodiversity, as it provides a deeper understanding of both patterns reconstructed from data and their underlying processes. In the simulations, we have experimented with two components of deep-time environmental change through deliberate isolation of these components and exclusion of many others that may have affected and shaped biodiversity. We have simplified habitat suitability by assuming that rivers and lakes host the same generic freshwater fish species. In future work, our findings should be integrated with the effects of changes in sediment provenance that affect the geochemistry and nutrient content of rivers, palaeoclimate evolution, changes in salinity and differences in species composition between upland and lowland rivers and lakes, among others. However, such holistic research is not possible without detailed understanding of the individual components. Our current study is therefore a first step into a mechanistic understanding of diversification and biodiversity evolution through deep geological time.

## Methods

### Biodiversity data

The data on freshwater fish diversity used in this study are derived from ref. ^4^. These authors collected occurrence data from 4,967 freshwater fish species in South America, calculated species richness values for all drainage sub-basins (490 basins; level 5 basins of the HydroBASINS database^49^) and performed a modelling procedure to correct for sampling bias. We corrected the raw sub-basin level data on species richness (Fig. 1b) for total habitat volume *H* available to fish within a sub-basin (Fig. 1c). To compute *H*, we made use of well-known scaling relationships between hydraulic variables that account for the fact that available habitat (that is, water volume per unit length of river) increases downstream as rivers increase in size. In particular, we made the following assumptions: (1) drainage density (that is, ratio between total length of the river network and the drained area) is constant in space, and hence the total river length *L*_tot_ within a sub-basin is proportional to the sub-basin area *A*_s_; (2) water velocity *v* is also constant in space^50^, hence water discharge *Q* (which by definition is equal to the product between *v* and the cross-sectional area *A*_C_ of the river); (3) *Q* is proportional to the drainage area *A* (ref. ^9^); and (4) within a sub-basin, all river reaches have the same *A*_C_. Under these assumptions, *H* is proportional to *A*_s_ × *A*, where *A*_s_ is proportional to *L*_tot_, and *A* is proportional to *A*_C_. As we are not interested in the exact value of *H*, but only in its spatial variation, we set *H* = *A*_s_ × *A*. We correct species richness (*S*) for *H* using *S* = *c* × *H*^z^, with coefficients (*c* = 0.089, *z* = 0.329, *P* < 0.01) obtained through a nonlinear least squared method.

### Palaeogeographic reconstruction

**Extended Data Fig. 6:**
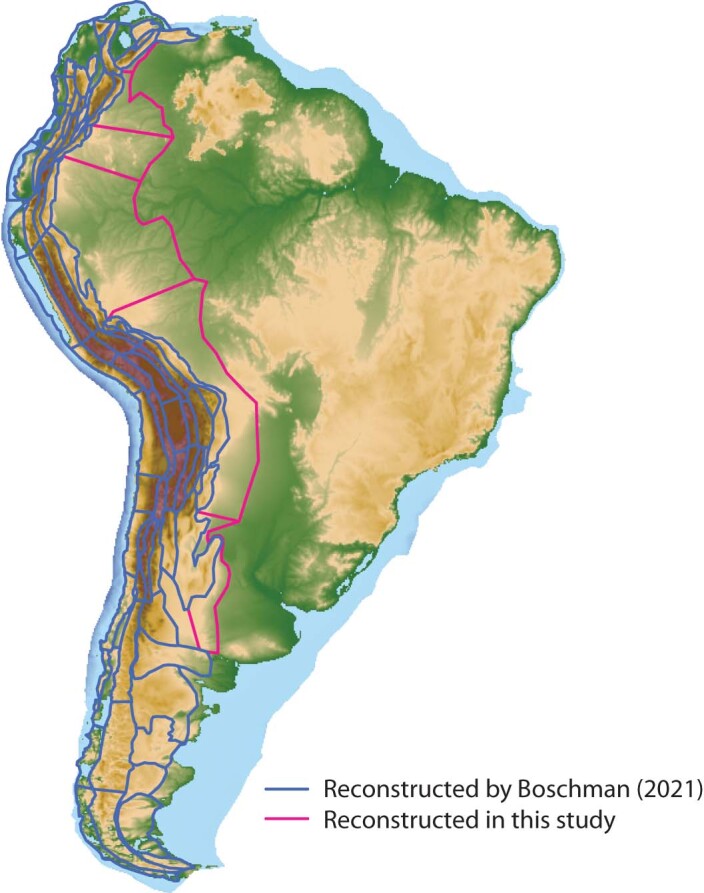
Area of reconstructed paleoelevational change. Map of South America, outlining the area in which elevation is reconstructed by the study of Boschman^21^, and this study.

**Extended Data Fig. 7:**
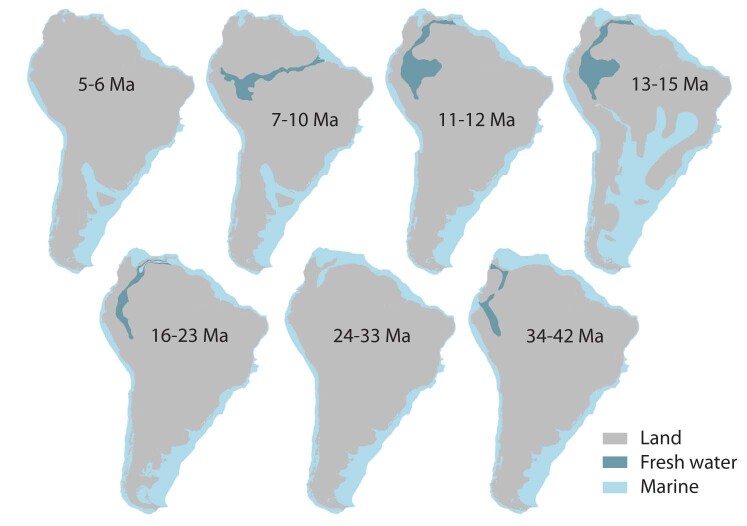
Reconstructed lakes and marine incursions. Outlines of lakes and marine incursions included in the reconstruction, based on the maps of Hoorn and Wesselingh^19^ for northern South America and Hernández, et al.^27^ for southern South America.

The dynamic landscape used as input in the biodiversity simulations is based on the reconstruction of Andean mountain building of ref. ^21^. This author^21^ compiled estimates of palaeoelevation and surface uplift for 36 individual domains in the Andes, and developed a reconstruction of 80 Ma of palaeoelevation, consisting of 80 palaeoDEMs in 0.1° resolution. This reconstruction is for the Andes only and does not include estimates of palaeoelevation of the rest of South America. We consider the relatively high elevations present along the eastern slopes of the Andes to be the result of the deposition of erosional material derived from the Andes during mountain building, and we reconstruct them as such, by lowering their elevation synchronously with the Andean mountain ranges to the west (Extended Data Fig. 6), using the topography reconstruction method of ref. ^21^. Furthermore, we assume that the current topography in the ancient Guianan and Brazilian shield areas was already present at 80 Ma, see the Drainage network reconstruction section for discussion on the implications of this assumption. We include the Miocene Pebas system and smaller preceding and following lakes, wetlands and marine incursions, based on the maps of ref. ^19^ for northern South America and ref. ^27^ for southern South America (Extended Data Fig. 7).

### River generation from palaeoDEMs

From the palaeoDEMs, we generated drainage networks via a method derived from the D8 algorithm^51^ and based on code developed for the R package OCNet^52^. First, drainage directions are established based on the steepest descent between neighbouring grid cells. A cell is not attributed a drainage direction if all neighbouring cells have equal (that is, the cell belongs to a flat area) or higher elevations (that is, the cell is an internal outlet). Second, drainage directions for flat areas are attributed by following the algorithm of ref. ^53^, which produces drainage directions away from higher terrain and towards lower terrain. Third, to solve for internal outlets, we apply an iterative procedure: for each internal outlet *o* (sorted by decreasing elevation), the contour of the region draining into *o* is determined, and the grid cell *c* on that contour with the lowest elevation is determined. The drainage path from *c* towards *o* is then reversed, so that *c* becomes the outlet of the catchment. If *c* borders a cell *c*’ that drains towards an outlet (either internal or along the coastline of the continent) whose elevation is lower than that of *o*, then a drainage direction from *c* to *c*’ is established; if more than one such cell *c*’ exists, the one draining towards the outlet with lowest elevation is selected. This procedure is repeated until all outlets coincide with the shoreline of the South American continent. To avoid the algorithm ‘finding’ the modern incised river valleys for each of the 80 time steps, we remove these present-day features from the landscape by lifting up all land lower than 100 m above sea level to 100 m. This approach proves robust as the algorithm produces river networks for the present day that match the modern configuration of South American rivers well (Fig. 2). Uncertainty in the reconstructed drainage networks is propagated from uncertainty in the palaeoDEMs, but as ref. ^21^ provides a best-estimate reconstruction only, this is not quantifiable. Nonetheless, the reconstruction used in this study provides a means to exploring plausible evolutionary scenarios given the currently available data on uplift history of the Andes. The obtained drainage networks include drainage directions for each individual 0.1° grid cell in South America, as well as a value for drainage area (that is, how many cells drain into the cell). The resolution of the river network used in the gen3sis simulations is controlled through this parameter river size (rs), thus using drainage area as a proxy for stream size^9^.

### gen3sis modelling

gen3sis^30^ is a modelling engine for the simulation of the evolution of biodiversity at the population level as a function of habitat change, through the mechanisms of dispersal, speciation and extinction. The model is mechanistic, meaning that speciation and extinction are not included as statistical parameters (that is, as model input), but instead occur, or do not occur, depending on changes in availability and connectivity of habitat. As a result, predicted patterns (that is, species richness) emerge dynamically from these underlying processes, rather than from statistical correlations with static spatial predictors (for example, climatic or geological). As with all modelling approaches, gen3sis serves as a simplified version of the real world, but the mechanistic nature of the eco-evolutionary processes and their dependency on habitat change and availability makes gen3sis particularly suitable to experiment with and test the role of palaeogeographic history in shaping biodiversity patterns. Habitat change is characterized by environmental conditions related to landscape evolution in a geographical framework. In this study, the landscape (in 0.1° resolution) is derived from the river generation algorithm. There are two possible states for a grid cell: suitable (representing lakes and rivers, produced by the river generation algorithm) or unsuitable (all other cells, representing land and marine habitat), and the state of a grid cell may change every time step. A river or lake is a connected series or group of suitable habitat cells and we do not include flow velocity or direction, water temperature, stream width and so on. The simulations are initiated with a single species that is present in all suitable habitat cells at 80 Ma, in line with the Late Cretaceous origin of the extant South American fish fauna^10,14,16^. Each (1 Ma) time step, species expand their range by dispersing to connected suitable habitat cells; the amount of range expansion is determined by a dispersal rate *d*. Modelled dispersal rates are several orders of magnitude smaller than empirical estimates, because on the scale of our 1 Ma model time steps, range expansion is not equal to maximum possible dispersal, as it is constrained by factors such as chance, competition and habitat suitability, which are not modelled explicitly, but implied. Similarly, modelled range expansion is equal in upstream and downstream directions. On short timescales (years), dispersal may be affected by flow direction, but on longer timescales, habitat suitability becomes the primary determinant in how much and in which direction species expand their ranges, as the chances of temporarily or accidentally overcoming barriers to dispersal in the upstream direction allowing fish passage become increasingly large^54,55^. For each time step in which two populations of the same species are disconnected, the populations build up units of ‘genetic distance’, and once this genetic distance reaches the divergence threshold (div), the two populations become two different species. A divergence threshold larger than 1 implies that gene flow can potentially undo the build-up of genetic distance: when isolated populations merge before reaching the divergence threshold, the genetic distance is reset gradually until reaching 0. A species goes extinct when all cells in its range change from suitable (river or lake) to non-suitable (land or marine) habitat. We use three input parameters: the minimum river size (rs), the divergence threshold (div) and the dispersal rate (*d*). River size rs controls the spatial resolution of the simulation and thus of speciation, which primarily affects the number of species generated by the model, but not species richness patterns (Extended Data Fig. 2). Therefore, we set this parameter to the finest resolution that allowed for a viable computation time (rs = 200). Similarly, div controls the temporal resolution of speciation and, when div > 1, also primarily affects the number of species generated by the model, but not species richness patterns. A div of 1, which means excluding gene flow in remerged populations prohibiting speciation, yielded different results (Extended Data Fig. 3). We set this parameter to the finest resolution that allowed for a reset of genetic distance upon merging of populations: div = *2*. The dispersal rate affected both the number of species generated by the model and the richness pattern of these species. We therefore simulated each scenario for a range of dispersal rates (*d* = 22.2, 33.3, 44.4, 55.5, 66.6, 77.7 and 88.8 km Ma^−1^, which roughly correspond to 2, 3, 4, 5, 6, 7 and 8 grid cells Ma^−1^, respectively; Extended Data Fig. 4). We compared model output to data by comparing values of simulated species richness with observed species richness per sub-basin, and calculating a Pearson’s *r* (Extended Data Figs. 2–4). In Fig. 3, we report the simulations in which the result of scenario C (the scenario that includes both surface uplift in the Andes and lakes in the Amazon basin, that is, that most accurately portrays the palaeographic history of South America) matches the data best (*d* = 5, that is, 55.5 km Ma^−1^; *r* = 0.61; Extended Data Fig. 4).

### Reporting summary

Further information on research design is available in the Nature Portfolio Reporting Summary linked to this article.

### Supplementary information

Reporting Summary

## Data Availability

All datasets required to run the analyses of this study are provided at the online repository Figshare: 10.6084/m9.figshare.19085603.v2. The HydroSHEDS^49^ database can be found at https://www.hydrosheds.org/.

## Code availability

All code required to run the analyses of this study is provided at the online repository Figshare: 10.6084/m9.figshare.19085603.v2. gen3sis (1.4) software can be downloaded from CRAN at https://CRAN.R-project.org/package=gen3sis.
