# Supplementary material for: Freshwater fish diversity in the western Amazon basin shaped by Andean uplift since the Late Cretaceous
Source: Nat Ecol Evol. 2023 Oct 19;7(12):2037–44. doi: 10.1038/s41559-023-02220-8 (PMC10697839; doi:10.1038/s41559-023-02220-8)
Supplement: Supplementary file 1 — Reporting Summary [file 41559_2023_2220_MOESM1_ESM.pdf]

nature portfolio

Corresponding author(s): Lydian Boschman

Last updated by author(s): 25/8/2023

Reporting Summary

Nature Portfolio wishes to improve the reproducibility of the work that we publish. This form provides structure for consistency and transparency in reporting. For further information on Nature Portfolio policies, see our Editorial Policies and the Editorial Policy Checklist.

Statistics

For all statistical analyses, confirm that the following items are present in the figure legend, table legend, main text, or Methods section.

n/a	Confirmed	
☒	☐	The exact sample size (*n*) for each experimental group/condition, given as a discrete number and unit of measurement
☒	☐	A statement on whether measurements were taken from distinct samples or whether the same sample was measured repeatedly
☐	☒	The statistical test(s) used AND whether they are one- or two-sided<br>*Only common tests should be described solely by name; describe more complex techniques in the Methods section.*
☒	☐	A description of all covariates tested
☒	☐	A description of any assumptions or corrections, such as tests of normality and adjustment for multiple comparisons
☐	☒	A full description of the statistical parameters including central tendency (e.g. means) or other basic estimates (e.g. regression coefficient) AND variation (e.g. standard deviation) or associated estimates of uncertainty (e.g. confidence intervals)
☐	☒	For null hypothesis testing, the test statistic (e.g. *F*, *t*, *r*) with confidence intervals, effect sizes, degrees of freedom and *P* value noted<br>*Give P values as exact values whenever suitable.*
☒	☐	For Bayesian analysis, information on the choice of priors and Markov chain Monte Carlo settings
☒	☐	For hierarchical and complex designs, identification of the appropriate level for tests and full reporting of outcomes
☐	☒	Estimates of effect sizes (e.g. Cohen's *d*, Pearson's *r*), indicating how they were calculated

Our web collection on statistics for biologists contains articles on many of the points above.

Software and code

Policy information about availability of computer code

Data collection	No new data was collected for this study.
Data analysis	All code required to run the analyses of this study is provided at the online repository Figshare: https://doi.org/10.6084/m9.figshare.19085603.v2. Gen3sis 1.4 software can be downloaded from CRAN at https://CRAN.R-project.org/package=gen3sis.

For manuscripts utilizing custom algorithms or software that are central to the research but not yet described in published literature, software must be made available to editors and reviewers. We strongly encourage code deposition in a community repository (e.g. GitHub). See the Nature Portfolio guidelines for submitting code & software for further information.

Data

Policy information about availability of data

All manuscripts must include a data availability statement. This statement should provide the following information, where applicable:

- Accession codes, unique identifiers, or web links for publicly available datasets
- A description of any restrictions on data availability
- For clinical datasets or third party data, please ensure that the statement adheres to our policy

All datasets required to run the analyses of this study are provided at the online repository Figshare: https://doi.org/10.6084/m9.figshare.19085603.v2. The HYDROSHEDS database can be found at https://www.hydrosheds.org/.

Field-specific reporting

Please select the one below that is the best fit for your research. If you are not sure, read the appropriate sections before making your selection.

☐ Life sciences ☐ Behavioural & social sciences ☒ Ecological, evolutionary & environmental sciences

For a reference copy of the document with all sections, see nature.com/documents/nr-reporting-summary-flat.pdf

Ecological, evolutionary & environmental sciences study design

All studies must disclose on these points even when the disclosure is negative.

Study description	This study comprises a modelling experiment, and a comparison between model output and previously published data. We combine previously published data on freshwater fish diversity (richness per sub-basin) in South America (Cassemiro et al. 2022), with a previously published reconstruction of mountain building in the Andes (Boschman, 2021), with a river generation algorithm (code provided), and a previously published mechanistic biodiversity model (gen3sis, Hagen et al., 2021). No new data is collected or analysed for this study.
Research sample	The data on freshwater fish diversity used in this study is derived from Cassemiro et al. 2022. These authors collected occurrence data from 4967 freshwater fish species in South America, and calculated species richness values for all drainage sub-basins (490 basins; level 5 basins of the HydroBASINS database).
Sampling strategy	n/a
Data collection	n/a
Timing and spatial scale	n/a
Data exclusions	n/a
Reproducibility	n/a
Randomization	n/a
Blinding	n/a

Did the study involve field work? ☐ Yes ☒ No

Reporting for specific materials, systems and methods

We require information from authors about some types of materials, experimental systems and methods used in many studies. Here, indicate whether each material, system or method listed is relevant to your study. If you are not sure if a list item applies to your research, read the appropriate section before selecting a response.

Materials & experimental systems

n/a	Involved in the study
☒	☐ Antibodies
☒	☐ Eukaryotic cell lines
☒	☐ Palaeontology and archaeology
☒	☐ Animals and other organisms
☒	☐ Human research participants
☒	☐ Clinical data
☒	☐ Dual use research of concern

Methods

n/a	Involved in the study
☒	☐ ChIP-seq
☒	☐ Flow cytometry
☒	☐ MRI-based neuroimaging
